# 3D Reconstructed Cyto-, Muscarinic M_2_ Receptor, and Fiber Architecture of the Rat Brain Registered to the Waxholm Space Atlas

**DOI:** 10.3389/fnana.2016.00051

**Published:** 2016-05-03

**Authors:** Nicole Schubert, Markus Axer, Martin Schober, Anh-Minh Huynh, Marcel Huysegoms, Nicola Palomero-Gallagher, Jan G. Bjaalie, Trygve B. Leergaard, Mehmet E. Kirlangic, Katrin Amunts, Karl Zilles

**Affiliations:** ^1^Institute of Neuroscience and Medicine 1, Research Centre JülichJülich, Germany; ^2^Institute of Basic Medical Sciences, University of OsloOslo, Norway; ^3^C. and O. Vogt Institute for Brain Research, Heinrich-Heine University DüsseldorfDüsseldorf, Germany; ^4^Translational Brain Medicine, Jülich-Aachen Research AllianceAachen, Germany; ^5^Department of Psychiatry, Psychotherapy, and Psychosomatics, RWTH Aachen UniversityAachen, Germany

**Keywords:** brain atlas, polarized light imaging, quantitative receptor autoradiography, histology, image registration

## Abstract

High-resolution multiscale and multimodal 3D models of the brain are essential tools to understand its complex structural and functional organization. Neuroimaging techniques addressing different aspects of brain organization should be integrated in a reference space to enable topographically correct alignment and subsequent analysis of the various datasets and their modalities. The Waxholm Space (http://software.incf.org/software/waxholm-space) is a publicly available 3D coordinate-based standard reference space for the mapping and registration of neuroanatomical data in rodent brains. This paper provides a newly developed pipeline combining imaging and reconstruction steps with a novel registration strategy to integrate new neuroimaging modalities into the Waxholm Space atlas. As a proof of principle, we incorporated large scale high-resolution cyto-, muscarinic M_2_ receptor, and fiber architectonic images of rat brains into the 3D digital MRI based atlas of the Sprague Dawley rat in Waxholm Space. We describe the whole workflow, from image acquisition to reconstruction and registration of these three modalities into the Waxholm Space rat atlas. The registration of the brain sections into the atlas is performed by using both linear and non-linear transformations. The validity of the procedure is qualitatively demonstrated by visual inspection, and a quantitative evaluation is performed by measurement of the concordance between representative atlas-delineated regions and the same regions based on receptor or fiber architectonic data. This novel approach enables for the first time the generation of 3D reconstructed volumes of nerve fibers and fiber tracts, or of muscarinic M_2_ receptor density distributions, in an entire rat brain. Additionally, our pipeline facilitates the inclusion of further neuroimaging datasets, e.g., 3D reconstructed volumes of histochemical stainings or of the regional distributions of multiple other receptor types, into the Waxholm Space. Thereby, a multiscale and multimodal rat brain model was created in the Waxholm Space atlas of the rat brain. Since the registration of these multimodal high-resolution datasets into the same coordinate system is an indispensable requisite for multi-parameter analyses, this approach enables combined studies on receptor and cell distributions as well as fiber densities in the same anatomical structures at microscopic scales for the first time.

## 1. Introduction

Virtual high-resolution multiscale and multimodal 3D models of the brain are essential tools to visualize and understand the complex structural and functional organization of the brain. To capture different aspects of brain organization, such as the long-range fiber tracts connecting different brain regions, intracortical connectivity, and differences in molecular compositions, complementary neuroimaging techniques should be utilized. In order to interpret and compare measurements derived from different experimental techniques, all brain data sets should be integrated into a standard reference space.

This integrative approach leading to a multimodal and multiscale brain model is a major challenge because of the enormous structural complexity of the brain. The different brain regions differ not only by their cytoarchitecture, i.e., the varying densities of cells between the different layers within and between brain areas, but also by the expression of neurotransmitter receptors and gene expression. This microstructural diversity leads to a segregation of the cerebral cortex and the subcortical regions into hundreds of well definable entities with complex spatial arrangements (Toga et al., 2006; Zilles and Amunts, 2010; Amunts et al., 2013; Amunts and Zilles, 2015). Moreover, the different entities are connected by long range and short range fiber tracts, which also show an enormous spatial complexity (Zilles and Amunts, 2012). Therefore, an accurate definition of the spatial positions of structural entities is an indispensable requirement, particularly for multimodal and multiscale data sets. This is far from trivial (Bjaalie, 2002), because it often requires the registration of data collected from different brains, with different spatial resolutions, different dimensions of methodically introduced structural deformations and artifacts, and structures of considerable intersubject variability. Therefore, a multiscale and multimodal analysis must be based on an integration of the various data in a common stereotaxic brain atlas framework.

The International Neuroinformatics Coordinating Facility (INCF) Digital Atlasing Project created such a standardized framework, i.e., Waxholm Space, that operates as a connection point between miscellaneous rodent brain data. The Waxholm Space (WHS) is a common open access (http://software.incf.org/software/waxholm-space) 3D reference space based on high resolution magnetic resonance imaging (MRI) data anchored in a standardized spatial coordinate system. It also supports infrastructure for data exchange. The WHS of the mouse brain (Hawrylycz et al., 2011) was extended amongst others with neuroanatomic atlases (Goldowitz, 2010), gene expression databases and MRI and diffusion tensor imaging (DTI) (Johnson et al., 2010). Papp et al. (2014) introduced and implemented the WHS atlas of the Sprague Dawley rat brain. The WHS rat brain atlas currently only contains high resolution MRI and DTI images, which served as basis for the delineation of the 79 major anatomical structures it depicts (Papp et al., 2014, 2015; Kjonigsen et al., 2015).

Aim of the present study was to complement the WHS rat brain atlas with information of cytoarchitecture, receptor expression and spatial orientation of fiber tracts. Thus, we processed entire postmortem brains of the Wistar rat for three different neuroimaging techniques: microscopic analysis of histological cell body stained serial sections for cytoarchitectonic analysis, *in vitro* receptor autoradiography to demonstrate muscarinic M_2_ receptor density distributions (Zilles et al., 2002; Zilles and Amunts, 2010), and 3D Polarized Light Imaging (PLI) for high resolution visualization of fiber tracts (Axer et al., 2011a,b). Imaging of the cell body stained sections enables a precise microscopical identification of cytoarchitectonically definable areas and the visualization of the spatial distribution of neurons. Quantitative *in vitro* receptor autoradiography is a well established technique to visualize the topographically heterogeneous distribution of neurotransmitter receptors, the key molecules of signal transmission. 3D PLI has been introduced recently and has opened up new avenues to analyze the complex architecture of nerve fibers and fiber tracts in postmortem brains at a microscopic resolution.

All of the above mentioned techniques require brain sectioning and mounting on glass slides, and this approach results in a loss of spatial alignment between neighboring sections. To obtain 3D brain models, the sections have to be aligned, the artificial deformations must be corrected, and 3D reconstructions must be performed. Therefore, it was necessary to improve currently available registration algorithms and adapt them to the specific requirements inherent to images obtained from receptor autoradiography and PLI. Finally, the nature of the data acquired in the present study also required the development of a novel registration strategy which enables integration of large scale high-resolution images of into the 3D MRI volume of the WHS rat atlas.

The here provided data complementing the Waxholm Space rat brain atlas will provide a multiscale and multimodal rat brain model enabling for the first time combined studies on receptor and cell distributions as well as fiber densities in the same anatomical structures at microscopic scales. Furthermore it will be publicly accessible through the Human Brain Project (HBP) portal, intended for multi-parameter analyses, refinement of the atlas labels, or further expansion via the proposed registration strategies.

## 2. Materials and methods

### 2.1. Tissue processing and image acquisition

#### 2.1.1. Tissue sectioning and blockface imaging

All animal procedures were approved by the institutional animal welfare committee at the Research Centre Jülich, and were in accordance with European Union (National Institutes of Health) guidelines for the use and care of laboratory animals. The brain of one adult male Wistar rat was used for the visualization of cell bodies and of muscarinic M_2_ receptors. It is referred to as the receptor brain. The brain of a second adult male Wistar rat, which we refer to as the PLI brain, was processed for the visualization of nerve fibers and fiber tracts. The receptor brain was immediately deep frozen in isopentane at −50°C and serially sectioned in the coronal plane at 20 μm thickness using a cryostat microtome (Leica Microsystems, Germany). The ensuing 1362 sections were thaw-mounted onto glass slides and organized in series of adjoining triplets of which one section was used for visualization of cell bodies, and the other two sections were processed for quantitative in vitro receptor autoradiography. The PLI brain was immersion fixed in 4% buffered formaldehyde. After two cryoprotection steps (10% glycerin for 3 days, followed by 20% glycerin for 14 days at +4°C), the brain was deep frozen in isopentane at −50°C and serially sectioned in the coronal plane at 60 μm thickness using the same cryostat microtome (Leica Microsystems, Germany). The 446 ensuing sections were placed on glass slides and stored at −80°C in airtight plastic bags until further processing. They were thaw mounted and coverslipped with 20% glycerin the day before image acquisition took place. During sectioning of both brains, blockface images of every section were taken with a CCD camera (AVT Oscar F-810 C, 3272 × 2469 pixels, 15 μm×15 μm, RGB) which was installed vertically above the cryostat, in order to obtain undistorted reference images. Spatial resolution in the z-direction was 20 μm for images obtained from the receptor brain, and 60 μm for images obtained from the PLI brain. A total of 1361 blockface images were taken for the receptor brain, and 446 for the PLI brain.

#### 2.1.2. Receptor brain

A total of 452 sections from the receptor brain were stained with a silver staining technique after (Merker, 1983). It results in a staining of all cell bodies, which is different from the widely used cresyl-violet stain of Nissl substance by its higher contrast and more intense visualization of cytoarchitecture. The sections processed for quantitative *in vitro* receptor autoradiography were used to demonstrate the densities (in fmol/mg protein) of two different receptor binding sites of the cholinergic muscarinic M_2_ receptor, i.e., the agonistic and the antagonistic binding site, according to previously published protocols (Zilles et al., 2002; Palomero-Gallagher et al., 2013). Sections were incubated with 1,7 nM 3H-oxotremorine-M (PerkinElmer, USA) or with 5 nM 3H-AF-DX 384 (PerkinElmer, USA) to visualize the agonistic and antagonistic binding sites of the M_2_ receptor, respectively. Binding assays were preceded by a preincubation in the respective buffer to eliminate the endogenous transmitters and finalized by a washing step. The labeled sections were exposed together with plastic scales of increasing and known radioactivity concentrations against beta-radiation (tritium) -sensitive films, which were developed after 15 weeks.

The ensuing 430 autoradiographs of the agonistic binding site of the M_2_ receptor as well as the 452 cell body stained histological sections were digitized using a high resolution camera (Zeiss) with an in-plane resolution of 5 μm×5 μm (4164 × 3120 pixels, RGB). Since each of these sections was 20 μm thick and sections had been organized into triplets, the resulting spatial resolution in the z-direction was 60 μm for both the digitized autoradiographs and the digitized histological sections. For further details of quantification of receptor density in fmol/mg protein and color coding see Zilles et al. (2002).

#### 2.1.3. PLI brain

The 446 sections from the brain cut at 60 μm were used to acquire 3D-PLI data reflecting the fiber architecture in gray and white matter regions (cf. Axer et al., 2011b; Dohmen et al., 2015; Menzel et al., 2015; Reckfort et al., 2015 for technical details). Briefly, 3D-PLI utilizes the optical birefringence of brain tissue, which is basically induced by the optical anisotropy of myelin sheaths wrapped around axons. By passing linearly polarized light through brain sections and by detecting the local changes in the polarization state of light, a 3D description of the underlying fiber architecture is derived. The imaging system used is a polarimeter. The sections were successively scanned with a large-area polarimeter (LAP), and subjected to an analysis workflow, which comprises calibration, independent component analysis, polarization analysis and calculation of fiber orientation maps (FOMs). FOMs are the fundamental data structure provided by 3D-PLI and have an in-plane resolution of 64 μm×64 μm, and, since each section was 60 μm thick, a spatial resolution in the z-direction of 60 μm. They contain a single 3D fiber orientation vector per voxel, that is interpreted as the spatial orientation of the fibers in this voxel.

### 2.2. 3D reconstruction

#### 2.2.1. 3D reconstruction of blockface images

Non-linear deformations introduced by brain sectioning, mounting and staining were corrected using blockface images as undistorted references for the spatial alignment of histological, autoradiographic and PLI images. Hence, in a first step the blockface images had to be 3D reconstructed. In short, the here applied robust and efficient reconstruction method consisted of a two-phase registration: a marker-based alignment of the blockface images and a refinement of the pre-reconstructed volume using 3D information. First, the coordinates of markers (circles) labeled on the microtome chuck were extracted. The centers of the circles of neighboring images were aligned to each other by means of a translation transformation. Processing all images leads to an almost smoothly reconstructed 3D stack of blockface images of the brain. However, this approach causes perspective errors due to the different heights of the sectioning plane and microtome chuck with the markers, and thus their different distances to the camera lens. Therefore, in the second part of the method the median along the z-direction of the marker-based reconstructed blockface volume was calculated to eliminate the outliers caused by perspective errors. The number of the images used by the voxelwise computation of this median volume can be specified by the radius of the median. In a next step, the marker-based reconstructed volume was aligned slice-by-slice onto the median volume using a translation transform estimated by an intensity based image registration algorithm with sum of squared differences as metric. By using this technique we took advantage of 3D information in an actually 2D slice-by-slice registration method. This led to an accurately aligned volume of blockface images that was an important reference to recover the spatial coherence of the non-linearly deformed sections corresponding to the blockface images. The procedure of 3D reconstruction of blockface images was introduced by Schober et al. (2015) with modified markers for the reconstruction. Finally, the reconstructed blockface volumes of the receptor and PLI brains were separated from the surrounding by means of a 3D watershed algorithm. The 3D reconstruction was carried out separately for images obtained from the receptor brain and for those of the PLI brain. Thus, we obtained two distinct blockface coordinate spaces (BCS): the BCS of the receptor brain (BCS^*R*^), and the BCS of the PLI brain (BCS^*PLI*^).

#### 2.2.2. 3D reconstruction of cyto- and receptor architecture images

**Figure 1:**
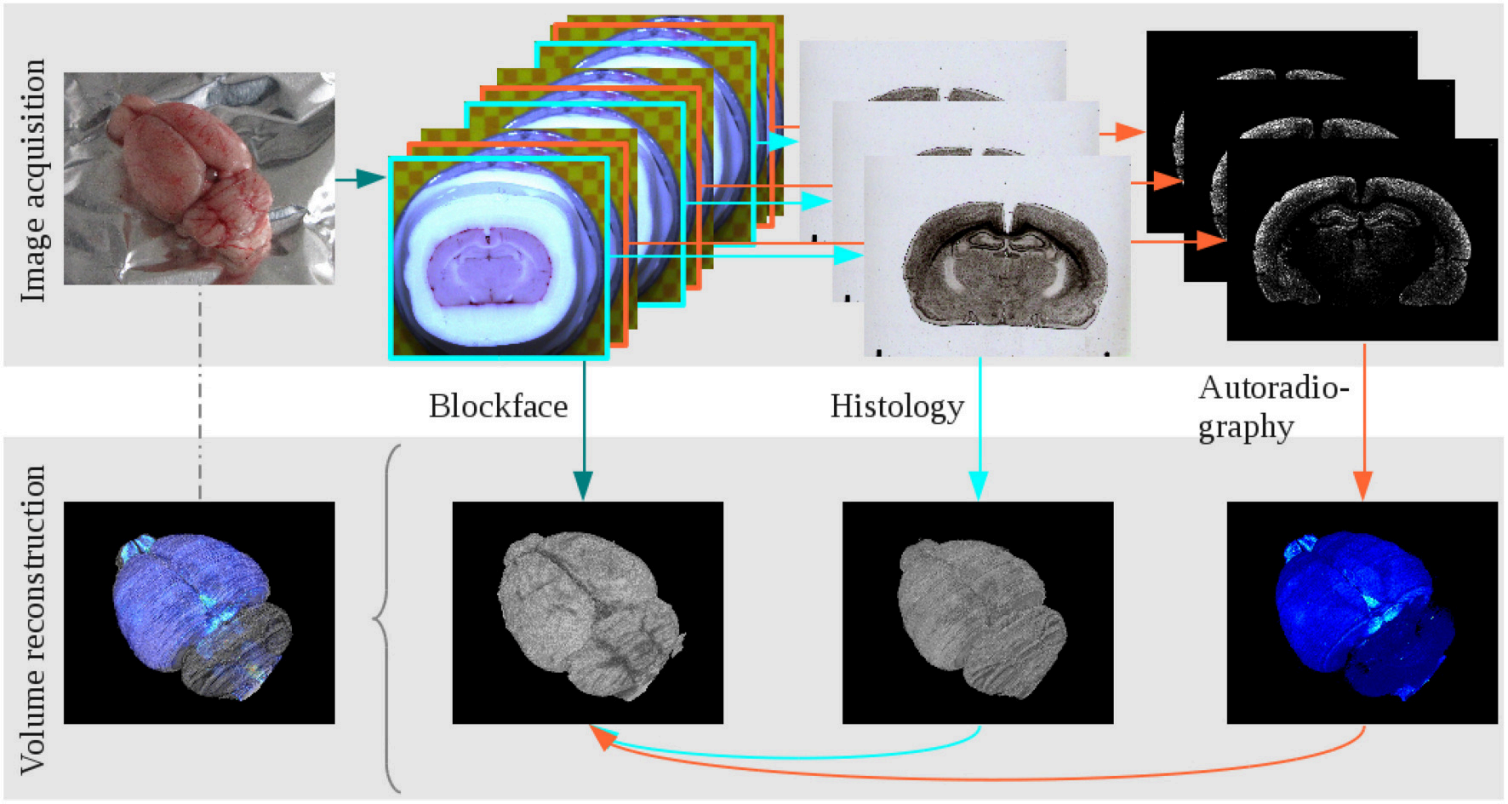
**The main steps of data acquisition and 3D reconstruction at the example of the receptor brain**. Image acquisition: The brain was removed from the head, blockface photographs were taken prior to sectioning, alternating sections were either used for cell body staining or receptor autoradiography. Volume reconstruction: The blockface sections were reconstructed, the 3D reconstructed blockface volume served as undistorted reference for the reconstruction of histological and autoradiographical sections. All modalities combined build a multimodal 3D model.

After reconstruction of the blockface volume of the receptor brain, each histological and autoradiographic image was aligned to its corresponding blockface image. Due to the highly different information each modality comprises (i.e., cell body distribution patterns vs. M_2_ receptor densities), it was necessary to establish different registration strategies. Each histological section was rigidly aligned with its corresponding blockface image. The centers of gravity of the brain tissue in blockface and histological image were calculated and superimposed. To determine the center of gravity, the separation of the brain tissue from the background is required. This was done by means of thresholding, extracting the largest connected component and morphological operations. After alignment of the centers of gravity a brute force optimizer tested all rotation angles with the sum of squared differences as metric. Details are described in Schubert et al. (2015). Reconstruction of the autoradiographic images is considerably more challenging due to the fact that receptors of a given type, in our case the muscarinic M_2_ receptor, are not necessarily expressed in all brain regions. Furthermore, when present, they can occur at very different concentrations throughout the brain. Thus, the range of gray values present in an autoradiographic image is much larger than that of a histological section. Therefore, the registration has to compensate for “empty regions,” i.e., regions without information in the images because that part of the brain does not express the receptor in question. First of all, an intra-stack registration matched consecutive autoradiographic images by means of a scale-invariant feature transform algorithm that detected characteristic points in the images based on their gradient information. Afterwards, these points were rigidly aligned by minimizing the Euclidean distance between them. With this pre-registered autoradiographic volume we were able to use a landmark based method to align the autoradiographic images to their corresponding blockface images. In every 30th image anatomical landmarks were manually set and the rigid transformation between these landmarks was calculated. Between these 30 images the transformations were interpolated and applied to the autoradiographic images (Huynh et al., 2015). Data acquisition and 3D reconstruction of the cyto- and receptor architecture are illustrated in Figure 1. At the end of this procedure, the 3D reconstructed histological volume and the 3D reconstructed M_2_ receptor volume were each in the BCS^*R*^.

#### 2.2.3. 3D reconstruction of fiber architecture images

The 3D reconstruction of the PLI data consists of two steps: a rigid slice-by-slice registration of the PLI images to the corresponding blockface images and a non-rigid refinement method. The first step is based on estimating a transformation of the PLI images to the corresponding image of the reconstructed blockface volume by image registration. To align the PLI images to the blockface images, the masks of the brain tissue of both data sets are required. For that, the reconstructed blockface volume was segmented by means of a 3D watershed algorithm and the PLI images were manually segmented. Using the segmented images the centers of gravity of the corresponding brain masks were calculated and aligned. Based on this initial transformation, an intensity based rigid registration was performed using mutual information as metric. The second step, the refinement, was done by means of a slice-by-slice B-Spline registration with sum of squared differences as metric and a grid size of 5 × 6. At the end of this procedure, the 3D reconstructed fiber volume was in the BCS^*PLI*^.

### 2.3. Data intergration into a reference space

#### 2.3.1. Waxholm space atlas

The WHS atlas of the Sprague Dawley rat brain is an open access atlas based on a high resolution MRI and DTI template in which both WHS and stereotaxic coordinates are defined. The T2^*^-weighted anatomical MRI (512 × 1024 × 512 pixels) with an isotropic spatial resolution of 39 μm was acquired ex vivo by means of a 7T small animal MRI system. Anatomical delineations in the atlas are based on image contrast observed in T2^*^-weighted images and diffusion tensor images. Technical details are described in Papp et al. (2014). The latest version of the atlas contains 79 structures with new and updated delineations of the hippocampal formation and parahippocampal region, as described in Kjonigsen et al. (2015). The atlas is available from the INCF Software Center (http://software.incf.org/software/waxholm-space-atlas-of-the-sprague-dawley-rat-brain).

#### 2.3.2. Data integration into the waxholm space atlas

In order to achieve an accurate analysis of the multimodal data sets, we aligned the atlas data to the coordinate space of each reconstructed data sets, i.e., to the BCS^*R*^ and the BCS^*PLI*^ by means of an advance image registration. In the literature several methods were proposed to integrate brain sections into 3D data volumes. Strategies which rely on successively increasing the degrees of freedom of the transformation demonstrated the best results. For instance, Dauguet et al. (2007) and Li et al. (2009) suggested a two step procedure consisting of a rigid transformation followed by non-linear transformations. However, this approach has several drawbacks, since a rigid transformation only aligns brain orientation, so that the brains after this transformation still differ in size and shape. Non-linear registrations work locally and, therefore, need well aligned volumes as starting point, which a rigid transformation cannot garantee. Lebenberg et al. (2010) added an affine transformation between the rigid and non-linear transformations. This step is also important for our data, due to the fact that the affine transformation is able to align the size and shape of the brains by scaling and shearing. However, since Lebenberg et al. (2010) not only registered a single mouse hemisphere, but also excluded the olfactory bulb and cerebellum, they were not confronted with the challenges posed by trying to register the whole brains. Therefore, modifications of their strategy are essential to enable accurate registration of structures such as the olfactory bulbus, or of even of the hemispheres, since they are independent of each other at levels rostral of (or caudal to) the corpus callosum. We used for all three transformations (rigid, affine and non-linear) a pyramidal method, i.e., a coarse to fine approach, to align initially large structures followed by aligning small and fine structures, whereas Lebenberg et al. (2010) only applied this multi-resolution approach to the affine transformation. Furthermore, we computed the similarity of the brains by means of Mutual Information, which is the best metric for multimodal registration tasks (Rueckert et al., 1999), in all three transformation steps, while Lebenberg et al. (2010) used Correlation Coefficient in the affine registration step. Finally, we studied the influence of the grid spacing used for the non-linear transformation to achieve best possible results for the whole brains, which was not tested by Lebenberg et al. (2010).

**Figure 2:**
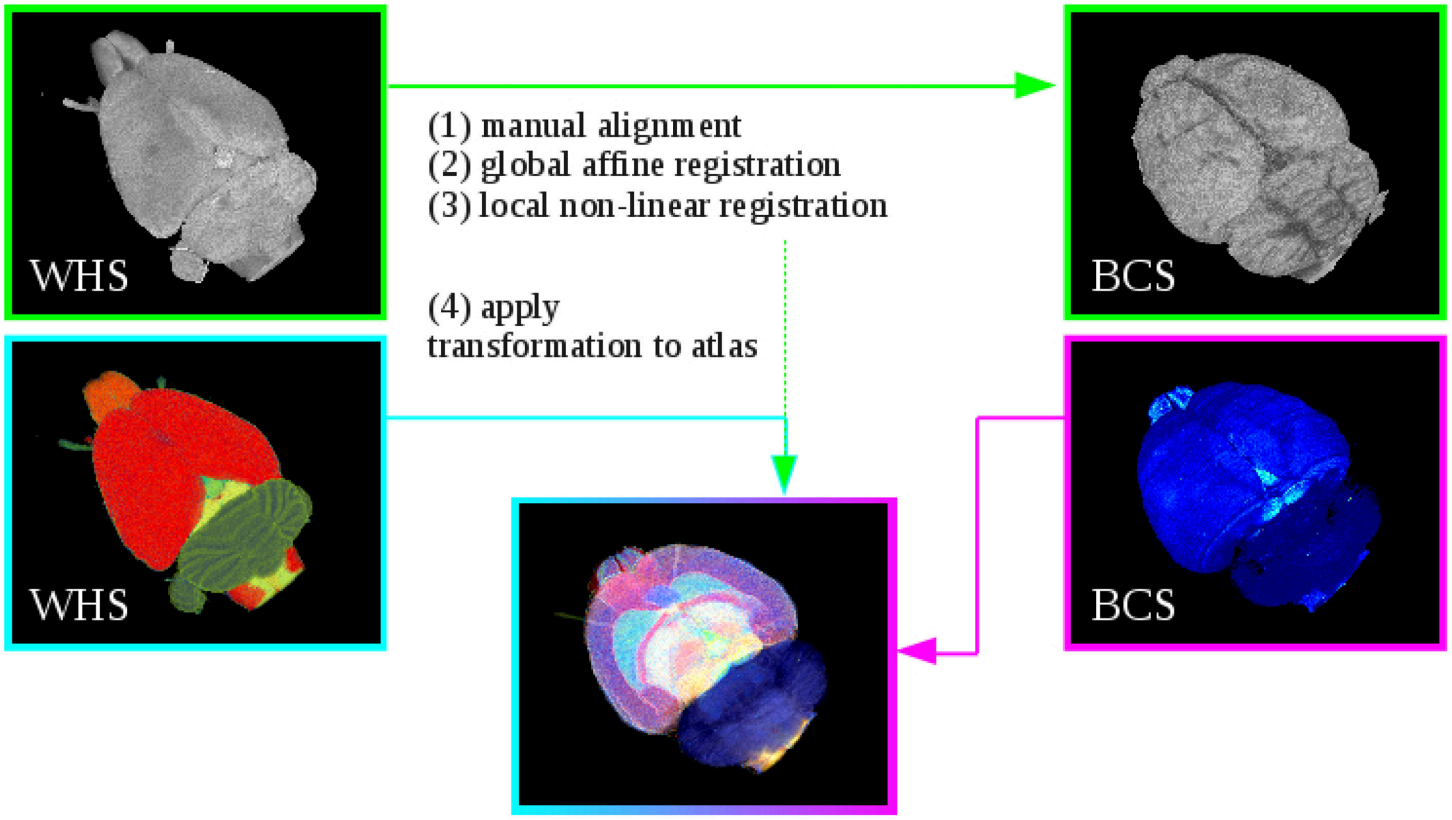
**Registration of Waxholm Space atlas with rat brain data in blockface coordinate space (BCS) at the example of the receptor brain**. Steps (1)–(3) performs the alignment of the WHS MRI to the blockface volume. Step (4) applies the estimated transformation from Steps (1)–(3) to the atlas. This yields the 3D atlas model of multimodal rat brain data.

The T2^*^-weighted atlas MRI was aligned to the respective reconstructed blockface volume. The estimated transformation was then applied to the digital atlas delineations. To compensate the variability between the atlas data and the rat brain data sets (i.e., the 3D reconstructed histological, M_2_ receptor and fiber volumes), the registration strategy consists of the successive steps explained below (Figure 2). Initially, image parts belonging to the rat brain of the MRI data set were separated from the rest of the head using the atlas template. All voxels in the MRI with their corresponding atlas label unequal to 0 were marked as “brain,” the other voxels were marked as background. The resulting masked MRI containing only the brain volume was used for the registration. Note that it was necessary to perform two separate registrations, since we have reconstructed blockface images in two separate spaces, namely BCS^*R*^ and BCS^*PLI*^. The procedure is the same in both cases and encompasses the following registration steps: The T2^*^-weighted MRI was manually aligned to a few selected images from the reconstructed blockface volume using the anchor a custom tool for affine registration of histological images to brain atlas space (Moene et al., 2011; Papp etal., 2016) in Navigator3. Then, the transformations were automatically propagated to the remaining images. These steps can be iterated with different parameters to prioritize specific boundaries or structures. Afterwards, the manually aligned MRI was automatically re-oriented to match the spatial orientation of the blockface volume. A global 3D affine registration initialized with the previously computed parameters was then optimized with normalized mutual information as similarity measure. To speed up the registration and prevent local minima a coarse to fine multiresolution approach was used which consisted of six levels of Gaussian smoothing pyramids. Finally, a 3D non-linear registration based on cubic B-Splines was used to refine the former parameters and local discrepancies. Again, normalized mutual information was chosen as similarity measure, and a pyramidal approach was used. The manual anchoring as well as the automatic 3D affine and non-linear transformations were directly applied to the atlas template with one exception. Instead of cubic B-Spline interpolation we used nearest neighbor interpolation to preserve accurate label boundaries and avoid gaps. We employed elastix (Klein et al., 2010) for the 3D registration. After registration to the respective blockface volumes, the resulting volume dimensions of the MRI and atlas template equal the dimensions of the blockface volume coordinate space: 996 × 1356 × 1361 pixels with a spatial resolution of 15 μm×15 μm×20 μm for the receptor brain (BCS^*R*^), and 588 × 723 × 446 pixels with a spatial resolution of 22 μm×22 μm×60 μm for the PLI brain BCS^*PLI*^.

#### 2.3.3. Evaluation of the registration results

Qualitative evaluation of the registration results in terms of anatomical accuracy was done by superimposing the aligned MRI and the blockface volume to compare the external borders and internal structures, as well as superimposing the atlas contours with the cyto-, muscarinic M_2_ receptor and fiber architecture.

Quantitative evaluation was performed by computing the quality of the alignment of atlas based segmented and receptor or PLI based segmented structures. Two structures which are defined in the WHS atlas were used: the pial brain surface, and the hippocampal formation. In our datasets different strategies were used to generate the pial surface contour and that of the hippocampal formation: The surface of the entire brains was segmented with a 3D watershed algorithm, and the hippocampal formation was manually delineated on the original images by two of the coauthors (NP-G and KZ). A comprehensive evaluation suggests the use of three uncorrelated measures that cover different aspects of the segmentation: an overlap measure (e.g., Dice coefficient), the Hausdorff distance and the average surface distance (Handels, 2000; Heimann et al., 2004). The Dice coefficient (DC) (Dice, 1945) assesses the overall overlap of the segments, it is sensitive to misplacement of the segments, but gives less weight to outliers. The average surface distance (ASD) and the Hausdorff distance (HD) determine the discrepancy of the surface of the segments. ASD is defined as the average error of all distances. A small ASD indicates a small error and variance between the segments. The HD returns the maximum distance between the segments, and therefore the maximum error. It is sensitive to outliers. The measurements were determined between an atlas based segmented structure *A* and the corresponding receptor or PLI based segmented structure *B*. The DC calculates the spatial overlap accuracy of two segmented structures *A* and *B*, whereby 0 is the result of disjunct segments and 1 is the result of a perfect agreement of the segments. With |·| denotes the number of voxels in the respective segmented structure, the DC is:

(1) $$\begin{array}{l} DC\left(A,B\right)=\frac{2|A\cap B|}{\left|A|+|B\right|} \end{array}$$

The ASD determines the minimal distance in mm of one segment to the other and vice versa. This value is 0 for a perfect registration.

(2) $$\begin{array}{l} ASD\left(A,B\right)=\frac{\underset{a\in A}{\sum }\underset{b\in B}{\min}d\left(a,b\right)+\underset{b\in B}{\sum }\underset{a\in A}{\min}d\left(b,a\right)}{\left|A|+|B\right|} \end{array}$$

The HD is defined as the maximum distance in mm of a segment to the nearest point in another segment and vice versa. A low HD indicates a good match.

(3) $$\begin{array}{l} HD(A,B)=\max(h(A,B),h(B,A))\text{ with }h(A,B)\\ \;=\underset{a\in A}{\max\;}\underset{b\in B}{\min}d(a,b) \end{array}$$

with the Euclidean distance *d* between point *a* and *b*.

Note, that the aim of this procedure was not to prove that our definition of pial surface or hippocampal formation is better than that of the WHS atlas. We only wanted to determine whether the overlap could be improved (i.e., differences could be reduced) with different algorithms.

### 2.4. Hard- and software

The processing was partially done using high-performance computing tools and supercomputing facilities of the Jülich Supercomputing Centre, Germany [Juelich Dedicated GPU Environment (JuDGE)], as well as the in-house Solaris computer cluster. Custom C++ software programs using ITK, elastix, OpenCV, MPI, OpenMP, QT and OpenGL performed the 3D reconstruction of the postmortem rat brains, the data integration into the WHS atlas, and furthermore the evaluation and the visualization of the results.

## 3. Results

The registration of the atlas MRI volume to the respective blockface volume was done in three subsequent steps: rigid, affine and non-linear B-Spline based registration. All three steps used the Adaptive Stochastic Gradient Descent approach for optimization. A multi-resolution registration with six levels was used to overcome local minima problems. The brain volumes were downsampled by a factor of 2 compared to the next resolution level. The similarity of the intensity values of blockface and MRI data was determined with the Mutual Information metric, which was specifically developed for multimodal data sets (Viola and Wells, 1997). As expected, the matching of brain structures from the different modalities improved considerably with increasing degrees of freedom of the transformations. Considerable differences were found after the rigid registration. The affine registration improved the matching, but still differences existed. The application of the non-linear registration led to a high matching quality. It took 39 min for the PLI brain (rigid 5 min 16 s, affine 5 min 16 s, B-Spline 28 min 30 s) and 66 min for the receptor brain (rigid 11 min 33 s, affine 12 min 8 s, B-Spline 42 min 18 s).

### 3.1. Qualitative evaluation

**Figure 3:**
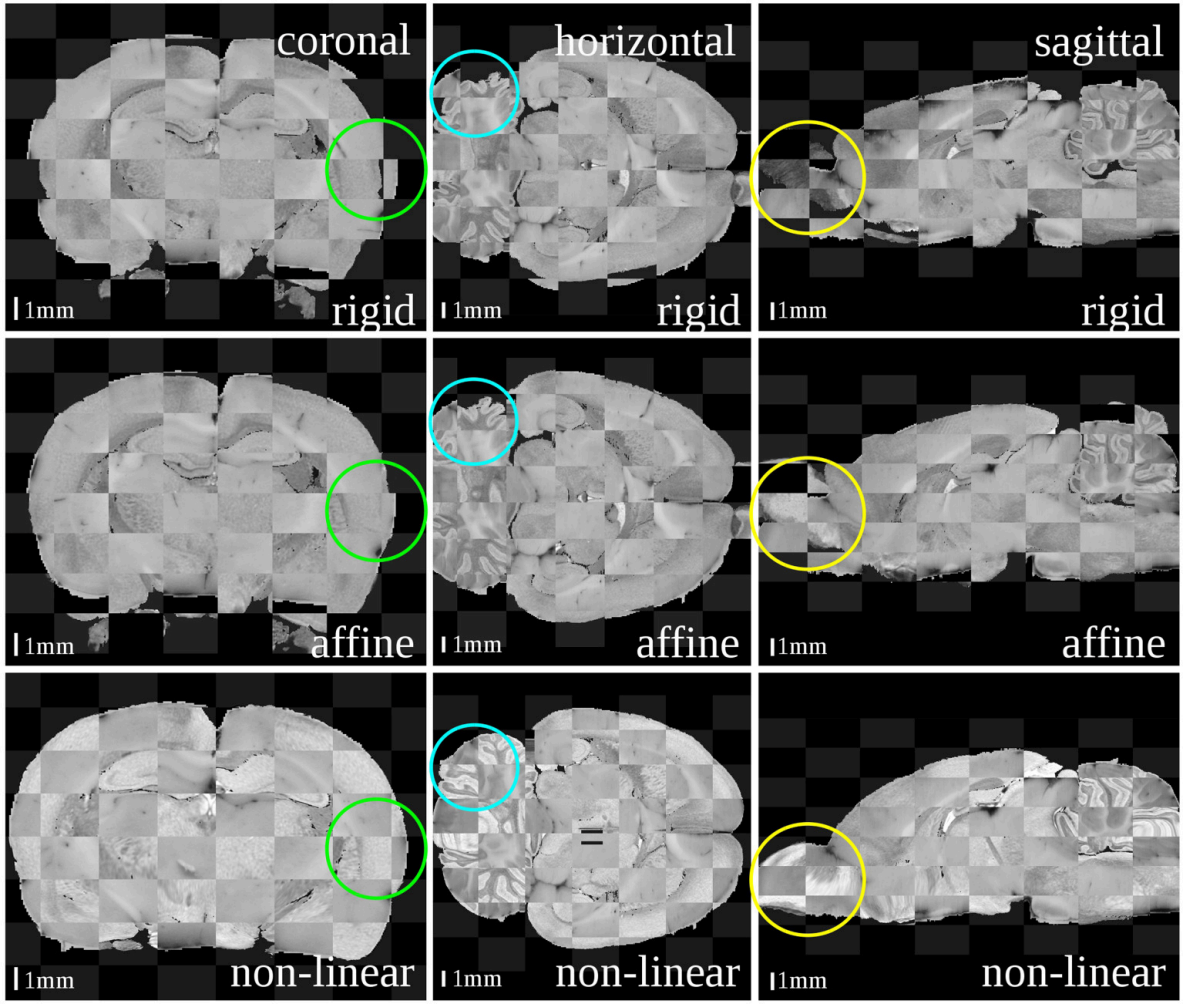
Checkerboard images of the blockface volume of the receptor brain and the T2^*^-weighted atlas MRI template after each registration step: rigid (first row), affine (second row) and non-linear (third row). Considering size and shape of the brain and especially its outer surface (green circles), cerebella (blue circles) and olfactory bulbs (yellow circles) the matching quality increased with increasing degree of freedoms of the transformations.

The results after each step are illustrated as checkerboard images of blockface and MRI volumes in three orthogonal views (coronal, horizontal and sagittal, c.f. Figure 3). Regarding the differences in size and shape of the brains, it was recognizable that these differences nearly disappeared from rigid, affine to non-linear registration transformation. Depending on the actual registration method, considerable to minor differences could be easily detected at three sites: the outer surface of the entire brain, the olfactory bulb and the cerebellum. The blockface volume of the entire brain was wider, that of the olfactory bulb was deflected and laterally displaced, and the cerebellum of the blockface volume was more flattened compared to the MRI volume. The difference between the outer surfaces (Figure 3, green circles) decreased after affine registration and disappeared after the non-linear registration. The position of cerebella (Figure 3, blue circles) and olfactory bulbs (Figure 3, yellow circles) considerably differed between blockface and MRI volumes. This could not be compensated by linear and global transformations. Only non-linear registration fitted these structures. In conclusion, rigid transformation sufficiently centered the brains, affine registration compensated differences in size and shape of the brains, and finally, non-linear registration aligned both small local mismatches and also large differences (c.f. olfactory bulb and cerebellum).

**Figure 4:**
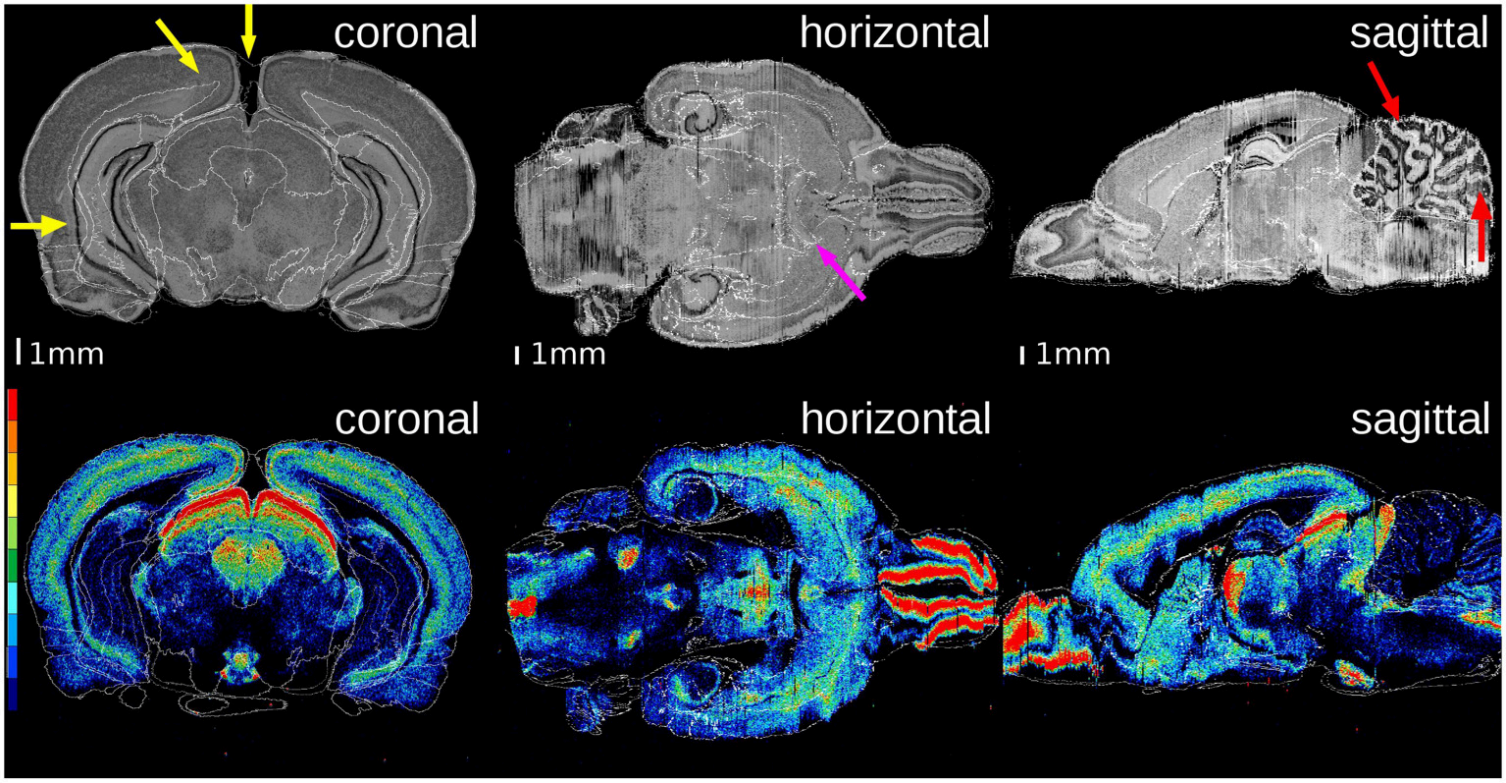
**Superimposition of the atlas structures (white contours) on the histological volume of the receptor brain (upper row) and on the M_2_ receptor density distribution volume (lower row)**. The color legend of the lower row denotes the distribution of M_2_ receptor densities (red: high, black: low). The overall matching quality is high, especially at the anterior commissure (magenta arrow). The gap between the hemispheres results in small mismatched boundaries (yellow arrows). The high differences of the cerebella in location and shape yields some small discrepancies (red arrows).

**Figure 5:**
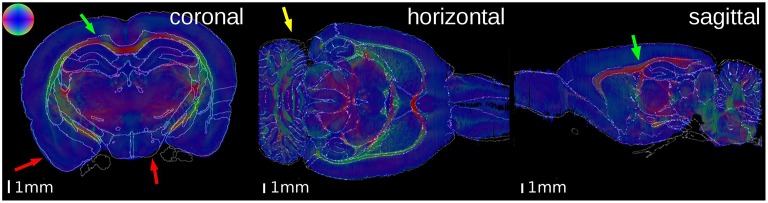
**Superimposition of the contours of the atlas delineations on the Fiber Orientation Maps (FOMs) of the PLI brain**. The color sphere indicates the direction of the fiber orientation. A high matching quality is observable, especially at the corpus callosum (green arrows). Some small discrepancies are visible at the outer surface of the brain (red arrows) and the cerebellum (yellow arrow).

The cytoarchitectonic, M_2_ receptor distribution, and fiber orientation volumes were superimposed with the atlas to demonstrate the quality of the match between the reconstructed and the atlas volumes (Figures 4, 5).

In both brains, the overall matching quality was high, particularly at the anterior commissure (Figure 4, magenta arrow) and the corpus callosum (Figure 5, green arrow). In the receptor brain, the artificial gap between the hemispheres was caused by the removal of the unfixed brain from the skull, which resulted in an anti-clockwise rotation and lateral displacement of the hemispheres and thereby local mismatches at the mesial cortical surface, the border between the retrosplenial cortex and the underlying white matter, and the medial protrusion of the neocortex in direction to the hippocampus (Figure 4, yellow arrows). The differences between the position and shape of the cerebella in the atlas and the reconstructions led to further mismatches (Figure 4, red arrows), which were not compensated by the registration. In the PLI brain, minor mismatches were found at the outer surface of the brain (Figure 5, red arrows) and the cerebellum (Figure 5, yellow arrow). The better match of the PLI brain is understandable, because the receptor brain was not fixed and therefore, more prone to distortions whereas the PLI brain was fixed before deep freezing.

### 3.2. Quantitative evaluation

The quality of alignment between the atlas and the reconstructed volumes was estimated by comparing the topography of the surface of the entire brain and of the hippocampal complex using three different measures introduced in Section 2.3.3. The surface of the entire brains was segmented with a 3D watershed algorithm. The atlas labels were used to extract the surface of the entire brain and hippocampal complex. The outer contour of the hippocampal complex was manually traced in the M_2_ and the FOM sections by experienced neuroanatomists. The hippocampal complex comprises the Cornu Ammonis regions 1, 2, and 3, the dentate gyrus and the subicular complex with the subiculum, presubiculum and parasubiculum. The hippocampal complex spans a wide portion of the brain in both the rostrocaudal and dorso-ventral directions, and can be used to demonstrate the registration quality for inner anatomical structures. Since the spatial location of the olfactory bulbs differed between MRI and blockface volumes, and caused far-reaching effects on the registration quality of many other brain structures, particularly at rostral levels, the quantitative evaluation was carried out with registrations with or without the olfactory bulbs. An important registration parameter was the spacing of the control points in the B-Spline grid, which indicates the flexibility of the transformation. A low spacing guaranties a high flexibility of the transformation.

**Figure 6:**
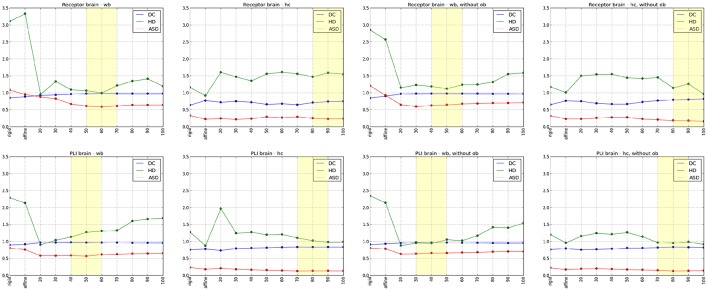
**Diagrams of the quantitative analyses of the Dice coefficient (DC), Hausdorff distance (HD) and Average Surface distance (ASD) from rigid, affine to non-linear transformations, whereby numbers 20–100 describes the flexibility of the grid (20 -high flexibility, 100-low flexibility)**. The upper row illustrates the registration results of the receptor brain and the lower row demonstrates the registration results of the PLI brain. Diagrams in the first column indicate the results of the analyses of the whole brains (wb), the diagrams in the second column indicate the results of the analyses of the hippocampal complex (hc). The last two columns show diagrams of the analyses of the wb (3rd column) and the hc (4rd column) after a registration excluding olfactory bulb (ob). The best results of the quantitative and the qualitative analyses are marked with yellow rectangles.

The results of the comparisons after each registration step using different spacing of the control points are shown in Figure 6. Using the Dice coefficient, the affine registration was sufficient to reach a high matching of the entire brain and the hippocampal complex well above 0.7, which is commonly accepted as a limit for a good match (Zijdenbos et al., 1994). However, the measures improved significantly after the non-linear B-Spline registration. The Dice coefficient and the average surface distance reached their optimum at middle flexibility of the transformation grid for the entire brain comparison. The Hausdorff distance reached an optimum at high flexibility. This is caused by the fact that the artificial displacements of the olfactory bulbs were compensated. However, this led to undesired transformations of brain structures at rostral levels. That was also reflected in the quantitative evaluation of the hippocampal complex. Here, the best results were achieved by the relatively less flexible affine registration (receptor brain), or after a non-linear registration at low flexibility levels (PLI brain). Comparing the results after registration without inclusion of the olfactory bulbs, the measures improved significantly for the hippocampal complex in the receptor brain. In contrast to the receptor brain the results between the registration with or without the olfactory bulbs did not considerably differ. In Table 1 the results are summarized.

**Table 1:** **The quantitative evaluation is measured by Dice coefficients (DC), Hausdorff distances (HD) and average surface distances (ASD) between receptor and PLI based and atlas based segmented structures, considering the whole brain and an internal structure, the hippocampal complex**.

			**Cyto- and receptor architecture**			**Fiber architecture**		
			**Rigid**	**Affine**	**Non-linear**	**Rigid**	**Affine**	**Non-linear**
Entire brain	Whole brain	DC	0.852	0.887	**0.981**	0.898	0.919	**0.976**
		HD	3.112	3.331	**1.000**	2.291	2.138	**1.139**
		ASD	1.085	0.952	**0.592**	0.810	0.767	**0.588**
	Hippocampal complex	DC	0.642	**0.776**	0.75	0.757	0.783	**0.84**
		HD	1.158	**0.92**	1.553	1.279	**0.877**	0.982
		ASD	0.332	**0.226**	0.238	0.235	0.186	**0.137**
Brain without olfactory bulb	Whole brain	DC	0.848	0.907	**0.978**	0.912	0.932	**0.971**
		HD	2.853	2.572	**1.124**	2.348	2.141	**0.946**
		ASD	1.208	0.929	**0.645**	0.808	0.788	**0.661**
	Hippocampal complex	DC	0.655	0.766	**0.824**	0.768	0.795	**0.841**
		HD	1.177	1.010	**0.961**	1.205	0.961	**0.956**
		ASD	0.323	0.235	**0.163**	0.224	0.174	**0.135**

*The Dice coefficient ranges between 0 and 1, 1 indicates full overlap. Lowest Hausdorff distance and average surface distance values indicate best alignments. The best results are labeled bold*.

## 4. Discussion

The study aimed at integrating multimodal (i.e., cytoarchitectonic, muscarinic M_2_ receptor distribution and fiber orientation data) and multiscale (i.e., mesoscopic resolution of blockface images and MR data of the 3D digital WHS Atlas, and microscopic resolution of sections) data in a common stereotaxic reference space. This was achieved by linear and non-linear registration. The qualitative and quantitative evaluations demonstrated a good matching of all data sets. We selected the whole brain, and additionally the hippocampal complex, that spans a wide distance within the brain in both the rostro-caudal and dorso-ventral directions, as examples to prove the quality of the methods of registration.

### 4.1. Methodic challenges

#### 4.1.1. 3D reconstruction

The 3D reconstruction of rodent brains is often carried out by means of rigid or affine registration transformations guided by blockface images (Ourselin et al., 2000; Lebenberg et al., 2010) or by volumes obtained from MRI (Li et al., 2009; Yang et al., 2012). We used blockface images for the reconstruction, due to the fact that they provide largely undistorted reference images of the brain sections. Furthermore, a reconstructed blockface volume is an excellent reference template, particularly if a real 3D volume (e.g., MRI volume) is missing or the resolution of the respective MRI volume is not appropriate (Schober et al., 2015). A particular challenge of the 3D reconstruction was the differential deformation of the brains inevitably caused by different tissue processing techniques. While the PLI brain was fixed and deep frozen before sectioning, the receptor brain was just deep frozen to maintain the receptor architecture. The fixation and deep freezing of the PLI brain introduces less deformations compared to the native brain size and shape than the deep freezing of the unfixed receptor brain (e.g., location of olfactory bulb). The sectioning procedure and mounting of sections from fixed brains also results in less deformations than that of sections from unfixed brains. To compensate these deformations, we performed, in addition to the common linear reconstruction strategies, non-linear transformations, which results in largely good reconstruction results. However, the anti-clockwise rotation of the hemispheres during the mounting procedure together with the more fragile nature of these unfixed cryostat microtome sections could not be completely eliminated at some sites (Figure 4, yellow arrows). A further challenge was the different information provided by the different modalities, e.g., “empty regions” (c.f. Section 2.2.2) in sections of the receptor brain. Therefore, particular reconstruction strategies were necessary for each modality, e.g., landmarks were interactively introduced in the receptor images, which was not required in the cytoarchitectonic and fiber tract images, because the latter images do not contain empty regions. In particular the strategy developed to solve problem of empty regions in receptor autoradiograhps represents a crucial step forwards in the reconstruction of future datasets coding for the regional and laminar distribution patterns of receptors, this is a recurrent problem, but the brain structures that do not express a certain type of receptors, or do so only at extremely low densities, vary considerably between receptor types.

#### 4.1.2. Data integration

Although many studies described the registration of 3D data, e.g., MRI volumes, to 3D atlases of rodent (Sergejeva et al., 2015) or human brains (Collins and Evans, 1997), only a few studies registered postmortem rodent brain sections to an MRI volume based atlas (Lebenberg et al., 2010, 2011; Abdelmoula et al., 2014; Sergejeva et al., 2015). Lebenberg et al. (2010) published the alignment of autoradiographic and histological data of one hemisphere of the mouse brain into a 3D digital MRI based atlas by means of a three step strategy containing rigid, affine and non-linear (elastic) transformations using the reconstructed blockface volume as intermediate modality between atlas and postmortem data. Abdelmoula et al. (2014) used a similar method to transfer mass spectrometry data into the Allen Mouse Brain atlas (Goldowitz, 2010) via affine and non-rigid B-Spline based registration. Sergejeva et al. (2015) identified anatomical landmarks in MRI, blockface or histological images for a landmark based affine registration of these data to the WHS rodent atlases (Johnson et al., 2010).

Since the 3D reconstructed receptor and PLI brains as well as the WHS Atlas brain slightly differed in size and shape of the entire brain and its inner structures, and the use of a non-linear registration is indispensable, we improved the three step registration strategy published in Lebenberg et al. (2010) by the constant use of a multi-resolution registration, i.e., application of a pyramidal method to all three transformation steps and of a similarity criterion (Mutual Information) as an optimal metric for multimodal registration tasks, and testing the influence of the grid spacing used by the non-linear transformation on the registration results. The combination of linear and non-linear transformations of the brains, and the use of the blockface volume as intermediate modality between fiber, cyto- and receptor architectonic and MRI data provided a maximal concordance of the brains. Rigid and affine transformations optimized the matching of the position of the different brains and compensated global shearing and scaling misalignments. Local structural adaptations were done with the non-linear B-Spline based registration. A crucial parameter was the flexibility of the B-Spline grid. With higher flexibility the algorithm generally works more accurately, but unrealistic deformations can be induced. This is illustrated by increasing the grid flexibility, which led to best overlap and distance results with the atlas brain (Figure 6). The overlap of the receptor or PLI brains and the MRI brain of the atlas was nearly perfect (Dice coefficients of 0.98 for M2 receptor brain; 0.97 for the PLI brain), but structures within the forebrain, the olfactory bulb and the cerebellum were unrealistically deformed. To overcome this problem, a lower grid flexibility was chosen, although this led to a lower overlap of the entire brains, with special focus in the region of the olfactory bulb. Since the position of the bulb in the receptor and the PLI brain does not reflect its natural position, but is extremely deformed by the necessary preparation steps for receptor autoradiography and PLI measurement, the sections through the bulb were excluded from the registration. This led to a much better overlap and improved distance measurements of the hippocampal complex, particularly in the receptor brain, and still to a good matching of the entire brains well above (Dice coefficient of 0.84 for both brains). Although the quantitative evaluation was based on automatically extracted contour in the case of the whole brain and on manually defined contours in the case of the hippocampus, a comparison of the results obtained for both structures reveals a high consistency. Likewise, although the quantitative evaluation of the matching of the hippocampal complex was based on independent delineations in the receptor, PLI and MRI brains by different experienced neuroanatomists, the results demonstrated high congruence of the different delineations of the hippocampal complex. This registration strategy was very effective in most brain regions. However, the remaining anti-clockwise rotation of the hemispheres in the fragile sections of the unfixed receptor brain and the mismatch between the neocortex and hippocampus in the center of the section (Figure 4) would only be compensated with an extremely high flexibility of the B-Spline grid. This would introduce large undesired artificial deformations in adjoining brain regions, which are biologically unrealistic.

### 4.2. Limitations and applications

We are aware that there are a series of putative limitations in the present study. One of them is that different sectioning thickness had to be used for processing of the receptor and PLI brains due to technical constraints. The PLI method requires fixation of brain tissue as well as a minimal section thickness in order to enable extraction of information concerning the direction of the fibers, and previous studies from our group have shown 60 μm to be an optimal thickness (Axer et al., 2011a,b). Quantitative in vitro receptor autoradiography requires usage of unfixed deep-frozen brains, since the method is based on the fact that the receptors to be visualized must maintain their ability to bind the radioactively labeled ligand present in the incubation buffer (Zilles et al., 2002). Unfortunately, it is technically not possible to obtain 60 μm thick sections tissue preprocessed in this manner, and, therefore, we used 20 μm thick sections. However, the different section thicknesses were accounted for during 3D reconstruction, so we do not think this poses a problem for the registration of our different image modalities to the WHS atlas. Quite the contrary, the methods developed here to overcome these differences in section thickness will facilitate future inclusion of multiscale data into the WHS rat atlas or the WHS mouse atlas.

Our cyto-, M_2_ receptor, and fiber architectonic datasets were obtained from adult Wistar rat brains, whereas the WHS atlas is based on an MRI scan of an adult Sprague Dawley rat (Papp et al., 2014, 2015; Kjonigsen et al., 2015), and the fact that brains from different rat strains have been used may also be viewed as a putative problem. However, this issue has been addressed in the past and is not thought to constitute a problem, since comparison of the cyto- and chemoarchitecture of the hippocampal formation in different rat strains has shown it to be a highly conserved brain structure (Kjonigsen et al., 2015).

A large variety of 3D digital atlases based on MRI or reconstructed histological or histochemical sections are available for rodent brains (Goldowitz, 2010; Dorr et al., 2008; Li et al., 2009, 2010; Johnson et al., 2010), nonhuman primate brains (Paxinos et al., 2000; Calabrese et al., 2015) and human brains (Hawrylycz et al., 2012; Shen et al., 2012; Amunts et al., 2013; Amunts and Zilles, 2015). Compared to the current available atlases the WHS (Hawrylycz et al., 2011; Papp et al., 2014) is an unique framework operating as a hub of an infrastructure connecting rodent brain data and reference spaces. To enrich this framework with cyto-, m_2_ receptor and fiber architecture provides a valuable extension to master analyses of the enormous structural complexity of the brain data.

### 4.3. Conclusion

**Figure 7:**
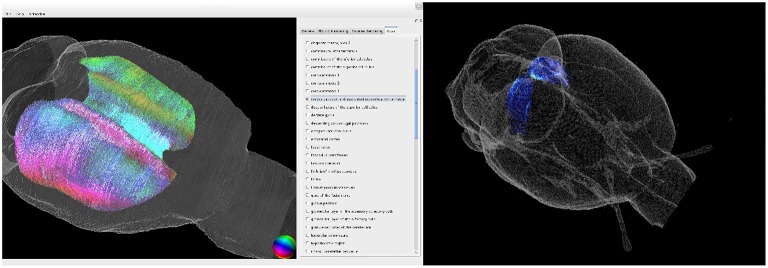
**3D visualization of the fiber architecture in the corpus callosum (left) and of the receptor architecture of the superior colliculus (right)**. Both structures were extracted using the delineations of the Waxholm Space atlas.

We developed a tool to register multiscale and multimodal rat brain data to the WHS atlas brain. It enables retrieval of detailed information of volume densities of cell bodies, of neurotransmitter receptor densities, and of fiber tract architecture and orientation in microscopically identified brain regions (c.f. Figure 7).

Therefore, our results considerably expand the data base of the WHS. Furthermore, the methods developed in the present study enable future integration of data of other modalities, which can further enhance the neuroscientific impact of the atlas. The 3D reconstructions of the cyto-, receptor and fiber architectonic images registered to WHS will be publicly accessible through the Human Brain Project (HBP) portal.

## Funding

This study was partially supported by the National Institutes of Health under grant agreement no. R01MH092311, by the Helmholtz Association through the Helmholtz Portfolio Theme “Supercomputing and Modeling for the Human Brain,” and by the European Union Seventh Framework Programme (FP7/2007-2013) under grant agreement no. 604102 (Human Brain Project) and by the Research Council of Norway (Norwegian Node of the International Neuroinformatics Coordinating Facility, project ID 214842).

### Conflict of interest statement

The authors declare that the research was conducted in the absence of any commercial or financial relationships that could be construed as a potential conflict of interest.
